# Prevalence, intensity and associated risk factors of soil-transmitted helminth infections among individuals living in Bata district, Equatorial Guinea

**DOI:** 10.1371/journal.pntd.0011345

**Published:** 2023-05-17

**Authors:** Gertrudis Ribado Meñe, Jean Claude Dejon Agobé, Juan Carlos Momo Besahà, Florentino Abaga Ondo Ndoho, Salim Abdulla, Ayôla Akim Adegnika

**Affiliations:** 1 Department of Environmental Education and Outreach, Faculty of Environment, National University of Equatorial Guinea, Malabo, Equatorial Guinea; 2 Ecole Doctorale Régional de l’Afrique centrale en infectiologie Tropicale, Franceville, Gabon; 3 Centre de Recherches Médicales de Lambaréné (CERMEL), Lambaréné, Gabon; 4 Institute of Tropical Medicine, University of Tubingen and Partner site Tubingen, German Center for Infection Research, Tubingen, Germany; 5 Department of health and safety, Bioko Island Malaria Elimination project in Equatorial Guinea, Malabo, Equatorial Guinea; 6 Direction of Public Health, Ministry of Health and Social Welfare, Malabo, Equatorial Guinea; 7 Department of intervention, Ifakara Health Institute, Dar–es- salaam, Tanzania; 8 Department of Parasitology, Leiden University Medical Center, Leiden, the Netherlands; 9 Fondation pour la Recherche Scientifique, (FORS) Cotonou, Benin; Emory University, UNITED STATES

## Abstract

**Background:**

Soil transmitted Helminths (STH) infections remain a public health concern worldwide, particularly in tropical and subtropical areas where these diseases are highly endemic. Knowing the prevalence and risk factors of the disease is crucial for efficient STH control strategies in endemic areas. The scarcity of epidemiological data on STH for Equatorial Guinea has motivated the decision to perform the present study.

**Methods:**

A cluster-based cross-sectional study was carried out in Bata district from November 2020 to January 2021. Stool samples were collected for the diagnostic of STH infections using Kato-Katz technique. Descriptive statistics was performed for determination of STH prevalence and intensity, while logistic regression models were used to assess the risk factors associated with STH infections.

**Results:**

A total of 340 participants were included in the study with a mean age of 24 years (SD = 23.7) and 1.2 female-to-male sex-ratio. The overall prevalence of any STH was 60% (95%CI: 55–65). The most prevalent species were *Ascaris lumbricoides* (43%, 95%CI: 37–48) and *Trichuris trichiura* (40%, 95%CI: 35–46). Intensity of infection were mainly light to moderate. A trend of association was observed between age and any STH infection (overall *p-value* = 0.07), with a significant difference observed between children aged 5–14 years as compared to those aged 1–4 (aOR 2.12; 95%CI: 1.02–4.43, *p-value* = 0.04), while locality was significantly associated with STH infection (overall *p-value*<0.001) with a higher odds observed for peri-urban area as compared to urban area (aOR 4.57; 95%CI: 2.27–9.60, *p-value*<0.001).

**Conclusion:**

Bata district is a high STH transmission area, where school-aged children and peri-urban areas are associated with a higher risk of any STH infection. This situation calls for a full implementation of the WHO recommendations for STH control; mass drug administration of anthelminthic twice a year to the whole population with great attention to school age children, and prioritizing peri-urban areas where safe water, improve sanitation, and hygiene education should be implemented to achieve a better control.

## Author summary

Soil-transmitted helminths (STH) remain a public health problem in endemic areas even though they are among the five preventive chemotherapy controlled neglected tropical diseases which benefit from regular mass distributions of antiparasitic drugs. Equatorial Guinea is known to be endemic for STH. However, epidemiological data on these infections needed for an effective control program are scarce or inexistent. For deworming implementation, passive strategies consisting of deworming of young children during vaccination campaigns and systematic treatment of children consulting at hospital for helminth-like symptoms are being implemented, as well as distribution of albendazole during lymphatic filariasis community mass treatment. In the present work, we provide current epidemiological data on STH infection in Bata district, which is the largest and most populated among the nineteen districts of Equatorial Guinea. We reported a high prevalence of STH infections of low to moderate intensity in the study areas, where school age children are more at-risk age group, and peri–urban area the more at-risk area. Our findings highlight the need to implement the WHO recommendations for control of STH infections in the country, such as mass drug administration of anthelminthic twice a year with great attention to pre-school-age children and school aged children. Health Education and other preventive measures such as WASH should be taken in consideration.

## Introduction

Soil transmitted-helminth (STH) infections are the most prevalent among the neglected tropical diseases (NTDs) [1–4], with 1,500 million people infected each year in 112 affected countries [5]. Four main species are responsible for the disease in humans; *Ascaris lumbricoides* with an estimation of 807 to 1.121 million people affected globally; *Trichuris trichiura* affecting 604 to 795 million people globally and hookworms (*Ancylostoma duodenale* and *Necator americanus*) infections affecting 576 to 740 million of people globally [6]. These parasites prevail in tropical and subtropical areas where ecological, socioeconomic, and environmental factors, together with the deficiency of hygiene enhance their transmission. At regional level, sub-Saharan Africa represents one of the most endemic regions in the world, supporting 40% of the worldwide burden [7,8].

STHs particularly affect children, hampering their physical growth and cognitive development. In this population, STH infections are known to cause malnutrition, micronutrient deficiencies, poor cognitive function with as consequence school absenteeism and low academic performance and wage earning potential [2,9]. Furthermore, STH infections has been reported to contribute to the host vulnerability to other endemic infectious diseases [10], such as human immunodeficiency viruses [11], malaria [12,13] and tuberculosis [14].

For the control of disease burden, the world health organization (WHO) recommends the implementation of some measures for population living in endemic areas, which include periodic mass drug administration (MDA) with albendazole (ABZ) or mebendazole (MBZ). The frequency of MDAs is determined by the baseline prevalence of STH infection in the community, particularly among school aged children (SAC). Treatment should be administered once a year if the prevalence is between 20–50% and twice a year if prevalence is 50% or above [15–18]. This highlights the importance of determining the STH prevalence of each endemic area. In addition to MDA and in order to control disease transmission, the WHO recommends the WASH (Water, Sanitation and Hygiene) strategy, which consists of providing safe water to at-risk population, adequate sanitation and hygiene education [15,19]. MDA campaigns are considered fundamental since they are cost effective targeting pre-school- (1–4 years) and school- (5–14 years) -aged children, and women of reproductive age (15 to 45 years old) all considered as more vulnerable [20,21]. However, in high-risk communities, infected adults and untreated children have been reported to act as reservoir of infection, raising the importance to take into account these population during MDA campaigns in such communities [2,9,20]. This strategy was supported in a mathematical model which demonstrate that the exclusion of adults in MDA campaigns in endemic areas contributes to the spread and re-infection of treated children [9].

Equatorial Guinea is a country located in central Africa region known to be endemic for STH [1,22]. The country is bordered to the north by the Republic of Cameroon, to the south and east by Republic of Gabon and to the west by the Equatorial Atlantic Ocean. With an area of 28,051.5 km^2^, Equatorial Guinea is constituted by two regions: the insular region and the continental region which comprises a large part of the country’s surface area. Administratively, both regions together are composed by eight provinces, 19 districts (18 districts before 2017) and 37 municipalities. Although the country is located in region known to be endemic for STHs, epidemiological data on STH infection are scarce for the country and those available are particularly old; about nine years and more [23–27]. Indeed, the main data was reported in 2008 through the national NTDs survey conducted by the Ministry of Health and Social Welfare (MoHSW) in all districts of the country, where the overall prevalence for any STH was 96%, and *T*. *trichiura* the most prevalent species with 91% prevalence followed by *A*. *lumbricoides* and Hookworms with 72% and 24% of prevalence, respectively [24]. On the STH control strategies, the government of Equatorial Guinea through the MoHSW and based on the first national epidemiological survey conducted in 2008, published in 2018 a strategic plan to control NTDs at national level in which the main STH strategies retained were MDA campaigns targeting children aged 5 to 15 years, provide safe water, and health education [28]. However, as indicated by the 2020 Expanded Special Project for Elimination of Neglected Tropical Diseases (ESPEN) report, no MDA have been implemented in the country until 2019 [29] mainly due to lack of funding [7]. Just passive strategies consisting of, deworming of young children during vaccination campaigns and systematic treatment of children consulting at hospital for helminth-like symptoms are being implemented. However, in a context of global development of the country and where the literacy rate among population older than 15 years is 94% [30], the 2011 Demographic and Health survey reported that 55% of the population has improved water sources with for instance, 21% of the population having public taps, 2% having taps at home and 11% using water wells. On sanitary facilities as well, 40% of the population reported having unshared toilets in improved conditions but only 8% discharged into sewers, while 12% of the population used shared sanitary facilities and only 1% of them discharged into sewers [31]. In that context, establishing current epidemiological data on STH infections in the country and more importantly identifying the main risk factors associated with infection could thus serve to develop a tailored guide for implementation of control strategies and to strengthen advocacy of a rapid scale up of interventions at national level. The objective of the present work was therefore to determine the current prevalence, intensity and risk factors associated with STH infections in Bata district, the largest district of Equatorial Guinea.

## Materials and methods

### Ethics statements

The study protocol was approved by the Equatorial Guinea National Ethical Committee in Malabo (CENGE; Nr Reg-2019-028). Administratively, the study received credentials from the public health direction of the ministry of health and social welfare (reference number: 56–150), the regional delegation of Bata of the ministry of health and social welfare (reference number: 2018), and the government delegation of Bata–Littoral from the Ministry of Interiors and Local Corporations (reference number 937). As study inclusion procedure, eligible participants filled out and signed the informed consent form and then were included. For illiterate adults who agree to participate, the informed consent form was filled out and signed by an impartial witness before the participant him/herself signed or thumb-printed the form. For participants less than 18 years of age who orally agree to participate, signed informed consent was obtained from parents or legal guardians before inclusion. The study was conducted in line with Good Clinical Practice principles of the International Conference on Harmonization [32] and the Declaration of Helsinki [33].

### Study area

**Fig 1:**
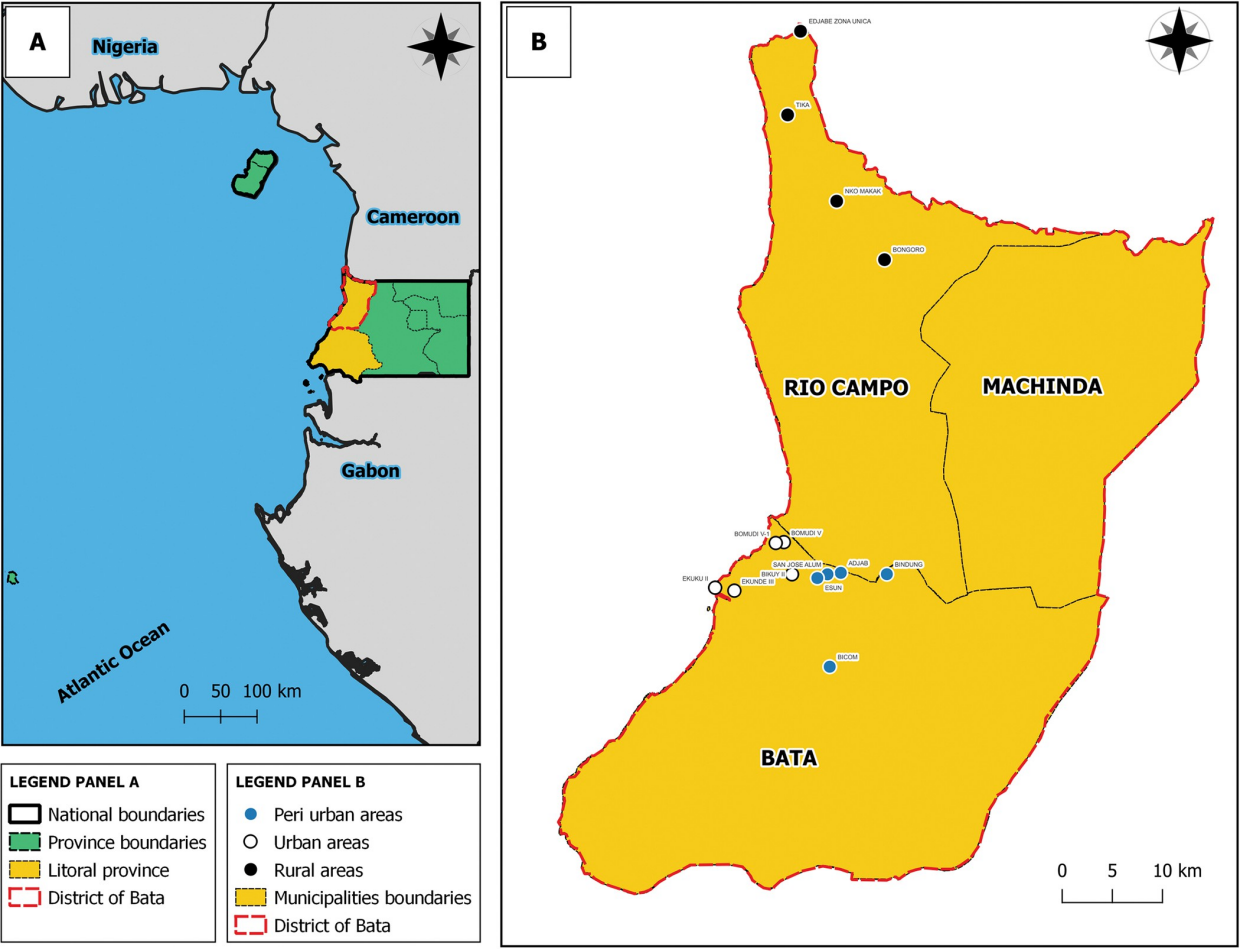
Map showing the geographical situation of Equatorial Guinea (Panel A) and neighborhood communities and village councils of the Bata district where the study was conducted (Panel B). The base layer for the country and national border shape was obtained from the Institut National de Cartographie (INC) of Gabon (https://data.bnf.fr/ark:/12148/cb15341899j); an open license database (https://data.bnf.fr/en/licence). QGIS version 3.28 was used to create the map.

This study was conducted in the municipalities of Bata and Rio Campo, in Bata district. Bata is the capital city of Littoral province, one of the eight provinces of Equatorial Guinea (Fig 1). Littoral province has the largest population in the country and is composed of three districts: Bata with 25% of country’s total population, Cogo, and Mbini. Bata district has three municipalities namely; Bata, the most populous; Machinda and Rio Campo [34]. Municipalities in Bata district constitute of urban, peri-urban, and rural areas. Urban areas are characterized by the presence of basic infrastructure available such as pipe water, internet, health, and education systems with easy access. In those areas, industrial economic activities are predominated, especially those of the secondary (manufacturing) and tertiary (services) sectors. Peri-urban areas are intermediate areas between urban and rural areas and were mainly located in peripheral regions of large cities. They are mainly made up of populations migrating from rural to urban areas in search of better living conditions and are therefore characterized by populations with low economic resources. Poor housing, unimproved latrines; precarious hygienic-environmental conditions are mainly predominant in peri-urban area while basic infrastructures such as sanitation systems for sewage, availability of safe drinking water, internet connection, health system, and school facilities are absent or scarce. Rural areas are areas located at great distances from urban centers and where predominate productive activities are of primary sector such as agriculture, livestock, and fishing. However, rural houses are better organized than in peri-urban areas. Bata district is known to be endemic for STH [7,8]. Indeed, a 94% overall prevalence of any STH was reported in the district in 2008, with *T*. *trichiura* as the most prevalent species [24].

### Study design and study population

We conducted a cross-sectional survey between November 2020 and January 2021. Participants of both sexes, from one year of age and above, residing in the study area at least 3 months before the start of the survey were eligible for the study.

### Sample size calculation

The sample size of the present work was calculated to determine the overall STH prevalence in the district of Bata where according to the 4^th^ population and housing census of 2015 [34], where 309,345 inhabitants are living. A previous survey conducted in the country in 2008 reported a 94% overall prevalence of STH in the same area [24]. Using the formula described elsewhere for sample size calculation for cross-sectional studies [35], 87 or more participants were needed to have a confidence level of 95% that the real prevalence is within ±5% of our measured value. As we planned to include our study population from the three main areas of the district of Bata; urban, peri-urban, and rural areas, we considered to include from each of those areas the calculated number of participants approximately equally distributed between neighborhood communities or village councils within each area. This gave us a total of 261 minimum participants to surveyed over the study area.

### Sampling procedure

A multi-stage cluster sampling technique was used for study sample selection. Bata district was our study area. At the first stage, we selected two (Bata and Rio campo municipalities) out of the three municipalities constituting the district of Bata. Bata municipality is composed by urban areas divided in neighborhood communities and peri-urban areas divided in village councils while Rio Campo municipality is composed by rural areas only divided in village councils. At the second stage of sample selection, five neighborhood communities in the urban areas and nine villages councils (five in the peri-urban areas and four in rural areas) were randomly selected using the list of neighborhood communities and villages provided by the Bata Littoral Government Delegation of the Ministry of Interior and Local Corporations. At the third stage, households were selected by convenience and from each household, a maximum of two eligible participants among those who were willing to participate were randomly selected and included in the study.

### Study procedures

After arriving at a selected house, we requested permission from the person in charge of the household. When permission was granted, the study objectives and participations conditions were explained to the house inhabitants in Spanish and when it was necessary in the community local language, and subject’s participation was requested. Among those who agreed to participate, eligible participants were included in the study. An identification code was assigned for each participant for confidentiality reasons. Socio-demographic data were recorded, and a short-standardized questionnaire was administered to all participants to collect data on the household conditions. For children aged less than nine years old, the head of family respond to their questionnaire as they were less likely to provide accurate answer. After administering the questionnaire, a clearly labeled plastic container for stool sample collection was provided to the participant who was invited to provide a morning stool sample the next day, or if not possible, the day after. Instructions on how to collect specimen were given to the participants prior to specimen collection. Fresh stool sample was collected in the morning, preserved in a thermal box and transported to Bata regional hospital laboratory in the following two hours of collection for analysis. Results were given back to all participants and to those found infected, MBZ 100 mg twice a day for three consecutive days was given free of charge.

### Stool sample processing

All stool samples collected were analyzed under a microscope using a Kato-Katz technique with a 41.7 mg mold, following the WHO standard procedure [18,36,37]. For each stool sample, two slides were prepared, and the two separate slides were read by two technicians. Preparation was examined under microscopy (10x and 40x power objective) after 30 minutes of slide`s preparation in a systematic manner. The eggs of each species counted was reported on a laboratory case report form. Participants was considered positive if at least one egg was found on any of the duplicate Kato-Katz thick smear slides [38]. STH infection intensity were calculated by multiplying the species specific average of egg counted from duplicate Kato Katz by factor of 24 [38].

### Statistical considerations

The statistical analysis aimed to describe the distribution of STH infections in the study population and the factors associated. Participants were considered as positive for any STH infections if found positive for at least one STH species. STH infection intensity was categorized as light, moderate or heavy based on WHO classification of intensity [39] as presented in S1 Table. Explanatory variables were sociodemographic characteristics, including age, sex, and location. Age was categorized into three groups based on the classification of population at risk of helminths infection in endemic communities as defined by the WHO; 1 to 4 years-old considered as preschool-age children (PSAC), 5 to 14 years-old considered as SAC and participant aged 15 years-old and more [39]. Similarly, we considered women aged 15 to 45 years-old as women of reproductive age (WRA). Education was classified as ‘no education’, ‘primary level’, and ‘secondary/university level’. As described early, locality was classified as ‘urban’, ‘peri-urban’, and ‘rural’.

The data was collected in a participant case report form and entered into Excel by a data clerk. A second data clerk double checked digitalized data. The clean database (S1 Dataset) was exported to R software version 4.1.1 for statistical analysis. Continuous variables were described as mean and standard deviation (SD) while categorical variables were described as frequencies or percentages and 95% confidence interval (CI). The difference between proportions was determined under the assumption that nonoverlapping CI indicated statistical significance. Independence between STH infection status and other categorical variables were tested using the Chi-squared test of independence (with Yates’ continuity correction for small sample size). Univariate and multivariate logistic regression model were used to identify factors associated with any STH infections. To fit the multivariate model, only indicators significantly related to outcome variable at a 25% level in univariate analysis were included. However, sex was forced in the model. Multicollinearity between variables was assessed using the variance inflation factor. Variable with the highest variance inflation factor was step by step removed from the model. Model with variance inflation factor less than five for all variables was retained as final model (Tables A and B in S2 Table). The significance level of all statistic tests was set at 5%.

## Results

### Participant’s enrollment flow

**Fig 2:**
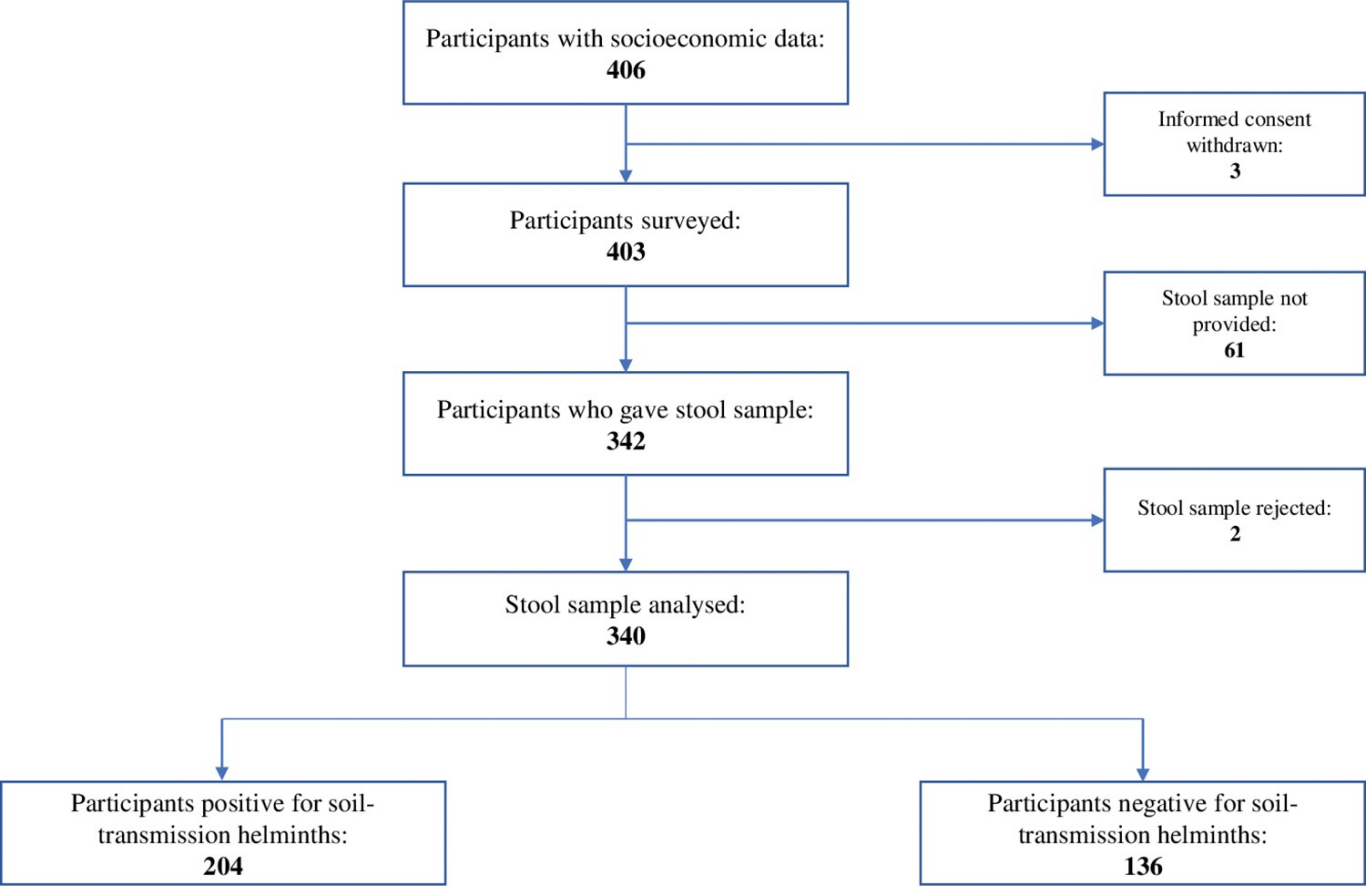
Study participant’s flow chart.

A total of 286 households were selected for a total of 406 participants evaluated for their eligibility to participate in the study. Of them, 403 participants were eligible and were invited to provide stool sample (Fig 2). Of them, 61 (15%) participants did not provide stool sample, while two (0.5%) samples were rejected because they were not correctly identified. This make a total of 63 participants considered as non-responders of which 54% was male and 63% were under 15 years old. Finally, samples from a total of 340 (84%) participants were retained and included for analysis.

### Study population characteristics

The mean age of the 340 study participants was 24 years (SD = 23.7). As presented in Table 1, participant age ranged between one and 86 years, with 42% of them aged from 15 to 86 years old and PSAC and SAC representing 19% (95%CI: 15%–23%) and 39% (95%CI: 34%–44%) of the study population, respectively. A total of 187 (55%) of the study population was female, with 1.2 female-to-male sex ratio. Women of reproductive age represented 21% (39/187, 95%CI: 15–27) of all females. Also, 41%, 35%, and 23% of the study population lived in urban, peri-urban and rural areas, respectively. A majority (55%) of them had primary school education level. Living conditions are presented in Table 2, 96% (326/340) of participants reported not to have sewerage system in their homes. For water source, a majority of 56% (189/340) of participants claimed to use tap water while 26% (89/340) of them used water from wells. The remaining 18% (62/340) use river water. Most study participants (49%, 168/340) were students; all individuals attending classes during the survey, irrespective of the educational level.

**Table 1:** Sociodemographic characteristics of study population.

		Study population		
		n	%	95%CI (%)
**Overall population**		**340**	-	-
**Age (mean, SD)**		**(24, 23.7)**		
**Age groups**				
	1–4	64	18.8	14.9–23.5
	5–14	132	38.8	33.7–44.3
	15–86	144	42.4	37.1–47.8
**Sex**				
	Female	187	55.0	49.5–60.3
	Male	153	45.0	39.7–50.5
	Ratio (F/M)	1.2	-	-
**Women of reproductive age**				
	No	148	79.1	72.5–84.6
	Yes	39	20.9	15.4–27.5
**Locality**				
	Urban	141	41.5	36.2–46.9
	Peri-urban	120	35.3	30.3–40.7
	Rural	79	23.2	18.9–28.2
**Education**				
	No education	91	26.8	22.3–32.0
	Primary	188	55.5	50.0–60.8
	Secondary/ University	60	17.7	13.9–22.3
**Daily occupation**				
	Student	168	49.4	44.0–54.8
	Farmer/ Fisher	65	19.1	15.2–23.8
	Unemployed	62	18.2	14.4–22.8
	Others	45	13.2	9.9–17.4

**Table 2:** Distribution of household characteristics among the 340 study participants.

		Study population		
		n	%	95%CI (%)
**House type**				
	Cement	128	37.6	32.5–43.1
	Wood	184	54.1	48.7–59.5
	Mixed	28	8.2	5.6–11.8
**House floor type**				
	Cemented	290	85.3	81.0–88.8
	Non-cemented	50	14.7	11.2–19.0
**Availability of toilets**				
	Present	325	95.6	92.7–97.4
	Absent	15	4.4	2.6–7.3
**Type of toilet**				
	Private /modern	156	48.0	42.5–53.6
	Communal latrine	64	19.7	15.6–24.5
	Traditional latrine / Hole	105	32.3	27.3–37.7
**Type of toilet floor**				
	Cemented	268	82.5	77.8–86.3
	Non-cemented	57	17.5	13.7–22.2
**Open defecation**				
	Never	103	30.3	25.5–35.5
	Sometimes	194	57.1	51.6–62.4
	Always	43	12.6	9.4–16.8
**Water source**				
	Tap	189	55.6	50.1–60.9
	Well	89	26.2	21.7–31.3
	River	62	18.2	14.4–22.8
**Sewage system**				
	Available	14	4.1	2.4–7.0
	Not available	326	95.9	93.0–97.6

### Prevalence of STH infection

The overall prevalence of STH was 60% (204/340; 95%CI: 55–65) with no significant difference for sex, (*p*-*value* = 0.69). Among WRA, the overall prevalence was 54% (95%CI: 37–70), with no difference as compared to their counterpart of other age (63%; 95%CI: 54–70). STH infections prevalence was higher in peri-urban areas (82%; 95%CI: 74–89), compared to urban (51%; 95%CI: 42–59) and rural (42%; 95%CI: 31–53) areas as shown in Table 3. The highest prevalence was observed in SAC (71%; 95%CI: 63–79) compared to adults (54%; 95%CI: 46–62) and PSAC (50%; 95%CI: 38–62). The distribution of STH overall prevalence per education level, occupation, and household characteristics is presented in S3 Table.

**Table 3:** Distribution per socio-demographic characteristics of STH infection among the study population.

		N	Any STH infection			*A*. *lumbricoides*				*T*. *trichiura*			Hookworm		
			n	%	95%CI (%)	n	%	95%CI (%)	n		%	95%CI (%)	n	%	95%CI (%)
**Overall population**		**340**	**204**	**60.0**	**54.6–65.2**	**145**	**42.6**	**37.4–48.1**	**137**		**40.3**	**35.1–45.7**	**13**	**3.8**	**2.1–6.6**
**Age**															
	1–4	64	32	50.0	38.1–61.9	24	37.5	26.0–50.5	17		26.6	16.7–39.3	1	1.6	0.1–9.5
	5–14	132	94	71.2	62.6–78.6	66	50.0	41.6–58.4	77		58.3	49.4–66.7	4	3.0	1.0–8.1
	15–86	144	78	54.2	45.7–62.4	55	38.2	30.3–46.7	43		29.9	22.7–38.1	8	5.6	2.6–11.0
**Sex**															
	Female	187	114	61.0	53.5–67.9	77	41.2	34.1–48.6	77		41.2	34.1–48.6	8	4.3	2.0–8.6
	Male	153	90	58.8	50.6–66.6	68	44.4	36.5–52.7	60		39.2	31.5–47.5	5	3.3	1.2–7.9
**Women of reproductive age**															
	No	148	93	62.8	54.5–70.5	65	43.9	35.9–52.3	63		42.6	34.6–51.0	6	4.1	1.7–9.0
	Yes	39	21	53.8	37.4–69.6	12	30.8	17.5–47.7	14		35.9	21.7–52.8	2	5.1	0.9–18.6
**Locality**															
	Urban	141	72	51.1	42.5–59.5	52	36.9	29.0–45.5	48		34.0	26.4–42.6	7	5.0	2.2–10.3
	Peri-urban	120	99	82.5	74.3–88.6	67	55.8	46.5–64.8	77		64.2	54.8–72.6	6	5.0	2.0–11.0
	Rural	79	33	41.8	30.9–53.4	26	32.9	23.0–44.5	12		15.2	8.4–25.4	0	0.0	0.0–5.8

The two most prevalent parasite species were *A*. *lumbricoides* and *T*. *trichiura* with 43% (95%CI: 37–48) and 40% (95%CI: 35–46) respectively, followed by hookworm 4% (95%CI: 2–7). *T*. *trichiura* (64%; 95%CI; 55–73) and *A*. *lumbricoides* (56%; 95%CI; 46–65) infections were more prevalent in peri-urban area, compared to urban (34%; 95%CI: 26–43 and 37%; 95%CI: 29–45) and rural (15%; 95%CI; 8–25 and 33%; 95%CI; 23–44) areas, respectively. With respect to the occupation, *T*. *trichiura* infection was most prevalent in students (52%; 95%CI; 45–60). Similarly, *T*. *trichiura* was more prevalent amongst SAC (58%; 49–67), compared to PSAC (27%; 95%CI: 17–39) and participants aged 15–86 years (30%; 95%CI: 23–38). Among WRA, the most prevalent species were *A*. *lumbricoides* (31%; 95%CI: 17–48) and *T*. *trichiura* (36%; 95%CI: 22–53), compared to hookworm (5%; 95%CI: 1–19). No difference was observed in the prevalence between WRA and their counterpart of other age for *A*. *lumbricoides*, *T*. *trichiura*, and hookworm infection.

### Intensity of STH infection

**Fig 3:**
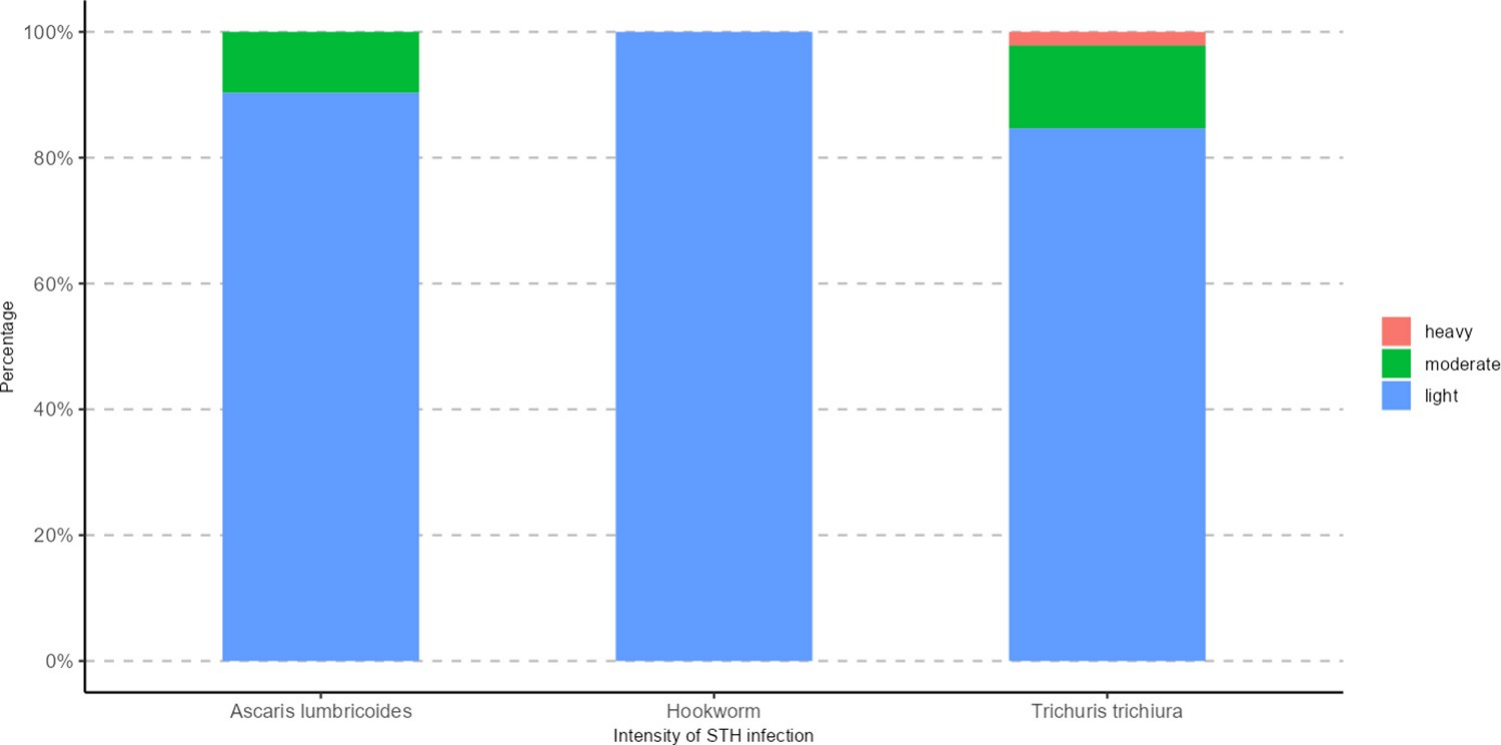
Distribution of the different intensity of the soil-transmitted helminth infections categories.

The median (IQR) egg count was 528 (IQR: 72–2256), 192 (IQR: 72–576), and 72 (IQR: 24–72) for *A*. *lumbricoides*, *T*. *trichiura* and hookworm infection, respectively. Fig 3 displays the proportion of the intensity of STH infections by species. STH infections were basically light intensity, with 100% for Hookworm infection, 90% for *A*. *lumbricoides* and 85% for *T*. *trichiura*. The prevalence of moderate intensity was 10% and 13% for *A*. *lumbricoides* and *T*. *trichiura*, respectively. Heavy intensity was observed for *T*. *trichiura* only, with 2%, and all of them are from peri-urban area.

### Distribution of STH co-infections

As presented in Table 4, 58% (95%CI: 51–65) of the 204 infected participants were mono-infected, with *A*. *lumbricoides* being the most prevalent with 51% (95%CI; 41–60), followed by *T*. *trichiura* with 45% (95%CI: 36–54) and Hookworm with 4% (95%CI; 2–10), respectively. Of the 86 (42%; 95%CI: 35–49) infected participants with co-infections, 94% (81; 95%CI: 86–98) of them were bi-infected, while the remaining 6% (5; 95%CI: 2–14) were infected with the three parasites. For bi-infection, the main representative combination was *A*. *lumbricoides–T*. *trichiura* co-infection with 96% (78, 95%CI; 89–99).

**Table 4:** Distribution of STH co-infection among infected study participants.

			Study participants		
			n	%	95%CI (%)
**Mono infection**			**118**	**57.8**	**50.7–64.6**
		*A*. *lumbricoides*	60	50.8	41.5–60.1
		*T*. *trichiura*	53	44.9	35.8–54.3
		Hookworm	5	4.2	1.6–10.1
**Co-infection**			**86**	**42.2**	**35.4–49.3**
	**Bi-infection**		**81**	**94.2**	**86.3–97.8**
		*A*. *lumbricoides–T*. *trichiura*	78	96.3	88.8–99.0
		*A*. *lumbricoides–*Hookworm	2	2.5	0.4–9.5
		*T*. *trichiura–*Hookworm	1	1.2	0.1–7.6
	**Triple infections**		**5**	**5.8**	**2.2–13.7**
		*A*. *lumbricoides–T*. *trichiura–*Hookworm	5	100	46.3–100.0

### Factors associated with STH infections

As presented in Table 5, a significant association was found at crude analysis between STHs infection and age (*p-value* = 0.005), locality (*p-value***<**0.001). As compared to PSAC, the SAC had 2.29 odds (95%CI: 1.27–4.19) to be found positive for STH infections while no significant difference was observed with the participants aged 15 and above (cOR = 1.04; 95%CI: 0.60–1.80). As compared to participants with no education, those with primary education level had higher odds, to be STH positive (cOR = 1.9; 95%CI: 1.14–3.18) while no difference was observed with those with secondary/university (cOR = 1; 95%CI: 0.52–1.93) education level. Regarding occupation status, farmer/fisher tend to have a lower odds of being infected with STH, compared to students (cOR = 0.56, 95%CI: 0.31–1.00, *p-value* = 0.05).

**Table 5:** Crude analysis of factors associated with any soil-transmitted helminth infections in the study population.

		Proportion of any STH infection (%)	Crude analysis		
			cOR	95%CI (cOR)	*p-value*
**Age**					**0.005**
	1–4	50.0	1		
	5–14	71.2	2.29	1.27–4.19	
	15–68	54.2	1.04	0.60–1.80	
**Sex**					**0.69**
	Female	61.0	1		
	Male	58.8	0.91	0.59–1.42	
**Women of reproductive age**					**0.30**
	No	19.0			
	Yes	5.6	0.69	0.34–1.42	
**Locality**					**<0.001**
	Urban	51.1	1		
	Peri -urban	82.5	4.52	2.58–8.18	
	Rural	41.8	0.69	0.39–1.20	
**Education**					**0.016**
	No education	51.6	1		
	Primary	67.0	1.90	1.14–3.18	
	Secondary /university	51.7	1.00	0.52–1.93	
**Occupation**					**0.007**
	Student	69.0	1		
	Farmer/fisher	55.4	0.56	0.31–1.00	
	Unemployed	50.0	0.45	0.25–0.81	
	Others	46.7	0.39	0.20–0.77	
**House type**					**0.43**
	Cement	59.4	1		
	Wood	58.7	0.97	0.61–1.54	
	Mixed	71.4	1.71	0.72–4.40	
**House floor type**					**0.75**
	Cemented	31.0	1		
	Non-cemented	29.0	1.10	0.60–2.07	
**Availability of toilets**					**0.28**
	Present	51.1	1		
	No present	2.2	1.88	0.63–6.90	
**Type of toilet**					**0.017**
	Private /modern	51.9	1		
	Communal latrine	60.9	1.44	0.80–2.63	
	Traditional latrine / Hole	69.5	2.11	1.26–3.58	
**Type of toilet floor**					**0.12**
	Cemented	27.6	1		
	Non-cemented	36.4	1.60	0.88–3.00	
**Open defecation**					**0.35**
	Never	54.4	1		
	Sometimes	61.9	1.36	0.84–2.21	
	Always	65.1	1.57	0.76–3.33	
**Water source**					**0.88**
	Tap	59.3	1		
	Well	60.7	1.07	0.64–1.81	
	River	61.3	1.15	0.64–2.10	
**Sewage system**					**0.74**
	Available	2.4	1		
	Not available	39.9	0.83	0.25–2.45	

**Fig 4:**
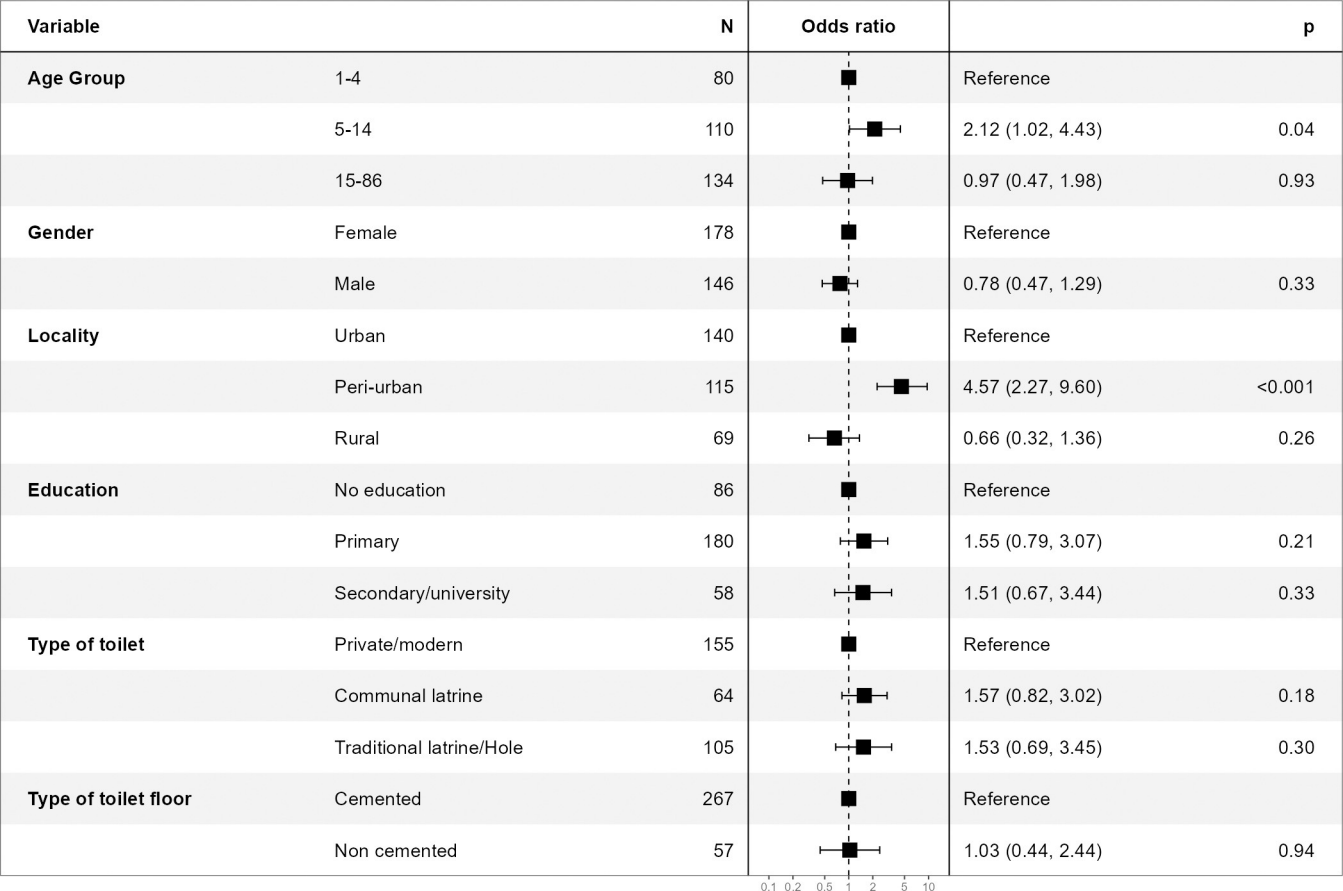
Multivariable analysis of factors associated with Soil-transmitted helminth infections.

The final model for adjusted analysis included age, sex, locality, education, type of toilet, and type of toilet floor. As presented in Fig 4, only a trend of a statistically significant association was observed for age (*p-value* = 0.07), while a strong statistically significant association was observed for locality (*pvalue* = <0.001). Compared to PSAC, the SAC had high odds to be infected with any STHs (aOR = 2.12; 95%CI: 1.02–4.43; *p-value* = 0.04), while no difference was observed with those aged 15 and above (aOR = 0.97; 95%CI: 0.47–1.98; *p-value* = 0.93). Comparing urban areas, there is a statistically significant high odds of being infected with STH with peri-urban area (aOR = 4.57; 95%CI: 2.27–9.60, *p-value*<0.001), while no difference in odds of being infected with STH was observed with rural area (aOR = 0.66; 95%CI: 0.32–1.36, *p-value* = 0.26).

## Discussion

Our results reveal a high prevalence of STH infections, classifying our study area as a high STH transmission area [10]. This finding agrees with the high prevalence previously reported in the same area through the National NTDs survey in 2008 [24]. However, our results are different to the moderate STH prevalence reported from a study on intestinal parasitism in an elementary school in Bata in 2008 [25]. This difference in result could be explained by the fact that the author conducted the survey in one primary school only. Our result suggests a persistence of infection transmission over time. Indeed, in Cameroon and Gabon, two border countries of Equatorial Guinea, a low STH infections prevalence has been reported after an MDA as compared to before intervention [40,41]. As those countries belong with Equatorial Guinea to the same geographical area, we can therefore assume that the lack of public health control measures implementation observed in our study area during almost the last 20 years [42] could explain the high prevalence reported. This high prevalence therefore expresses an urgent need of implementation of MDA campaigns as recommended by the WHO in order to reduce the STH prevalence in particular and the disease morbidity in general that we can assume to be high in our study area [43].

We investigated the distribution of STH species in our population and found that the most predominant species are *A*. *lumbricoides* and *T*. *trichiura* with moderate prevalence for each, as compared to the prevalence of hookworm species which was found to be low. This result could indicate a lower transmission of hookworm infection in the study area, compared to *A*. *lumbricoides* and *T*. *trichiura*. Natural environmental conditions such as tropical climate, soil type mainly sandy typical in Bata district [34], and local temperatures [44] combined with the absence of sewage system, habits of no processing drinking tap water could explain the finding. In our study population for instance, 96% of study participants reported not having a sewage system connected to their houses. In addition, knowing that *T*. *trichiura* and *A*. *lumbricoides* eggs can resist several years in the soil, and considering that *A*. *lumbricoides* and *T*. *trichiura* transmission is fecal-oral, in absence of adequate sewage system, feces and sewage containing parasite eggs can persist in the environment, contaminating water, soil and get infective [19,37,45] and favor the transmission of these two species in particular. In the same vein, investigating the STHs co-infection in our study population, the level of prevalence we reported for each species could explain the pattern of co-infection we found. Indeed, *A*. *lumbricoides* and *T*. *trichiura* were both involved in 96% of co-infections. This pattern of STH distribution corroborates with those published in the central Africa region in 2009 [1] but opposes the finding from Democratic Republic of Congo where the most prevalent species where hookworm, followed by A. *lumbricoides* and *T*. *trichiura* [46]. Our finding could indicate that *A*. *lumbricoides* and *T*. *trichiura* are the species with highest transmission even in the sub-region, as compared to hookworm infection. Indeed, *T*. *trichiura* was reported as the most abundant STH infections in Cameroun and Gabon, followed by *A*. *lumbricoides* and hookworms [47,48], mainly because ABZ and MBZ used in those areas for the control of STH are known to be less effective for the treatment of *T*. *trichiura*. In Bata district, we found similar level in prevalence for *A*. *lumbricoides* and *T*. *trichiura* which could highlight the absence of MDA or few implementations of large-scale campaign of ABZ or MBZ in the country, as well as absence of WASH strategy.

Although the prevalence of STH infection was high among the study population, the intensity of those infections was basically light to moderate. This finding corroborates with what is reported by some authors in area with either moderate [49] or high [50] STH prevalence and where the intensity of the infections was mainly light to moderate. We hypothesize that the low proportion of heavy infection intensity we observed could be due to the combined effect of deworming young children during vaccination campaigns and the “systematic” administration of anthelminthic treatment to individuals with helminth-like symptoms consulting in hospitals, as it is empirically observed in the country. Indeed, heavy intensity infection should be more frequently associated with clinical symptoms and will therefore lead patients to seek treatment. Treatment of mainly patients presenting symptoms could therefore contribute to the reduction of intensity of infection and morbidity in the community.

Assessing the risk factors of STH infections in our communities, we found locality as a main risk factor. As compared to urban or rural areas, peri-urban areas were found to be more at risk of STH infections. Indeed, we found a significant highest prevalence of *A*. *lumbricoides* and *T*. *trichiura* in peri-urban area compared to urban and rural areas. Our result corroborates with the finding reported from a georeferenced estimates of STH prevalence worldwide were *A*. *lumbricoides* and *T*. *trichiura* infections were found most prevalent in peri-urban areas [51]. However, a study conducted in South West Cameroon found rural areas as more at risk locality [52]. We observed that peri-urban areas with inadequate housing conditions, and poor socio-environmental hygiene conditions that increase the risk of STH infection as disease transmission [51]. This finding indicates that the MDA campaigns implemented in the peri-urban areas must be supported by the full implementation of the WASH strategies in order to be efficient.

We found SAC to be more at risk of infection, compared to other age groups which corroborates with many other studies [10,20,53]. This is mostly due to their playing habits, less hygiene practices, and lack of WASH in schools and at home. Although SAC were more at the risk, we found a high prevalence of STH infections in adults which can have an implication on the control program in the country. Indeed, based on the WHO recommendations, the periodic MDA should target PSAC and SAC, WRA, and adults with at-risk activities such as farming, selling agricultural products at the market, and less hygiene measures practices [54]. Therefore, not taking into account all adult groups in MDA strategies, particularly in endemic areas where less or non STH control strategies were previously developed, can make them to serve as reservoir of infection in the communities [47], and make further control programs ineffective. We therefore advocate inclusion of adults in MDA campaign in our communities where STH prevalence is high if we want the country to control the disease first and then to reach the WHO 2030 targets for STH elimination as public health problem [43,55]. Education of SAC on STH transmission and prevention could boost the prevention of the disease in the community.

We used Kato-Katz technique for the STH infection diagnostic. Although this technique is mostly used in population-based prevalence survey, it is known to be more sensitive for *A*. *lumbricoides* and *T*. *trichiura*, and less for hookworms [18,56,57]. This could thus contribute to the low prevalence and low intensity for hookworm infection we reported, even if similar prevalence (4%) was already reported in 2012 in Bata but among HIV population [11]. Despite all, Kato Katz technique is considered by the WHO as the gold standard diagnostic method for the detection and quantification of STH intensity [18]. It has been demonstrated that sensitivity of Kato Katz and other microscopic method also depends on the infection intensity. We reported here a moderate or low infection intensity, which could lead to an underestimation of the prevalence we found but not on the conclusion we drawn on the level of disease transmission in the area. Despite the limitation of our diagnostic method technique, the present study offers a picture of the situation of STH infection in Bata district, classified as high STH infection transmission area. Such information can therefore serve as a guide for the orientation and implementation of control strategies [19,53]. In addition, the present study was carried out in only one out of 19 districts. Our conclusion could be therefore limited to the Bata district. However, as Bata district is the most populous district of the country, we could anticipate that our finding could drive the implementation of the STHs control programs in the country. Also, the sample size include in the present study could appears low, particularly to assess the risk factors of the disease and to compare prevalence between modalities of variables. However, on one hand the 95% confidence interval we reported for the prevalence we found may support the consistency of the sample size we included, and on the other hand the statistically significant results we found with the actual sample size could assume a strong association between those risk factors and STH infectious status, particularly when looking at the high STH prevalence reported in the study area. Finally, no information was collected on the use of anthelminthic in the study population months prior the samples collection, could influence the prevalence and intensity we reported in the present study, but cannot affect our conclusion.

## Conclusions

The present study reveals that Bata district is a high STH transmission area, where age and locality are the main risk factors of infection in the population. Our results indicate a need of the implementation of two rounds of MDA campaigns per year which should include populations of all age, but with particular attention to SAC and population living in peri-urban areas where WASH concept should be implemented. For a tailored and efficient interventions, future research on knowledge, attitudes, and practices of population regarding STH infections could be contributive.

## Supporting information

S1 Checklist STROBE checklist. (DOC)

S1 Dataset Database. (XLSX)

S1 Table Word Health Organization classification of the intensity of *Ascaris lumbricoides*, *Trichuris trichiura*, and hookworm infections. (DOCX)

S2 Table Table A. Assessment of the multi-collinearity using the Variance Inflation Factor (VIF) in the initial final model of the multivariable analysis Table B. Assessment of the multi-collinearity using the Variance Inflation Factor (VIF) in the revised final model of the multivariate analysis.
(DOCX)

S3 Table Distribution of STH infection per education level, occupation, and household characteristics among the study population. (DOCX)

## References

[1] Hotez PJ, Kamath A. Neglected tropical diseases in sub-Saharan Africa: Review of their prevalence, distribution, and disease burden. PLoS Neglected Tropical Diseases. 2009; 3:2–11. doi: 10.1371/journal.pntd.0000412 19707588 PMC2727001

[2] Ozano K, Dean L, Macpherson E, Theobald S, Halleux C, et al. Discussion paper the gender dimensions of neglected tropical diseases. UNDP-Led Access and Delivery Partnership. 2019. Available from: https://adphealth.org/upload/resource/2523_ADP_Discussion_Paper_NTDs_211119_web.pdf

[3] Hotez PJ, Aksoy S, Brindley PJ, Kamhawi S. What constitutes a neglected tropical disease? PLoS Neglected Tropical Diseases. 2020;14: 1–6. 10.1371/journal.pntd.0008001 PMC6991948 31999732

[4] Álvarez-Hernández DA, Rivero-Zambrano L, Martínez-Juárez LA, García-Rodríguez-Arana R. Overcoming the global burden of neglected tropical diseases. Therapeutic Advances in Infectious Disease. 2020; 7:1–3. doi: 10.1177/2049936120966449 33178435 PMC7592315

[5] Pullan RL, Smith JL, Jasrasaria R, Brooker SJ. Global numbers of infection and disease burden of soil transmitted helminth infections in 2010. Parasites & Vectors. 2014. doi: 10.1186/1756-3305-7-37 24447578 PMC3905661

[6] Bethony J, Brooker S, Albonico M, Geiger SM, Loukas A, Diemert D, et al. Soil-transmitted helminth infections: ascariasis, trichuriasis, and hookworm. Lancet. 2006. doi: 10.1016/S0140-6736(06)68653-4 16679166

[7] WHO. Regional Strategy on Neglected Tropical Diseases in the Who African Region 2014–2020. 2014. Available from: https://www.afro.who.int/sites/default/files/2017-06/regional-strategy-on-neglected-tropical-diseases-in-the-who-african-region-2014%E2%80%932020%20%281%29.pdf

[8] AU. Draft Continental Framework on the Control and Elimination of Neglected Tropical Diseases in Africa by the Year 2030. 2020. Available from: https://espen.afro.who.int/system/files/content/resources/SA26699%20_E%20Original_Continental%20framework%20on%20NTDs.pdf

[9] Ásbjörnsdóttir KH, Means AR, Werkman M, Walson JL. Prospects for elimination of soil-transmitted helminths: Current Opinion in Infectious Diseases. 2017;30: 482–488. 10.1097/QCO.0000000000000395 PMC7680933 28700363

[10] Crompton DWT, World Health Organization. Preventive chemotherapy in human helminthiasis: coordinated use of anthelminthic drugs in control interventions: a manual for health professionals and programme managers. 2006. Available from: https://apps.who.int/iris/handle/10665/43545

[11] Elobo MR. Caracterización de parásitos intestinales asociados a la infección por VIH en Guinea Ecuatorial. PhD Thesis, Universidad de Zaragoza. 2012. Available from: https://zaguan.unizar.es/record/9898/files/TESIS-2012-133.pdf

[12] Degarege A, Veledar E, Degarege D, Erko B, Nacher M, Madhivanan P. *Plasmodium falciparum* and soil-transmitted helminth co-infections among children in sub-Saharan Africa: A systematic review and meta-analysis. Parasites and Vectors. 2016; 9. Available from: doi: 10.1186/s13071-016-1594-2 27306987 PMC4908807

[13] Dejon-Agobé JC, Zinsou JF, Honkpehedji YJ, Ateba-Ngoa U, Edoa JR, Adegbite BR, et al. *Schistosoma haematobium* effects on *Plasmodium falciparum* infection modified by soil-transmitted helminths in school-age children living in rural areas of Gabon. PLoS Neglected Tropical Diseases. 2018; 12:1–17. doi: 10.1371/journal.pntd.0006663 30080853 PMC6095623

[14] Cadmus SI, Akinseye VO, Taiwo BO, Pinelli EO, van Soolingen D, Rhodes SG. Interactions between helminths and tuberculosis infections: Implications for tuberculosis diagnosis and vaccination in Africa. PLoS Neglected Tropical Diseases. 2020. doi: 10.1371/journal.pntd.0008069 32498074 PMC7272205

[15] WHO. Fifty-fourth World Health Assembly. Ninth plenary meeting. 2010. Available from: https://apps.who.int/iris/bitstream/handle/10665/78794/ea54r19.pdf

[16] WHO. First WHO report on neglected tropical diseases: working to overcome the global impact of neglected tropical diseases. World Health Organization. 2010. Available from: https://apps.who.int/iris/handle/10665/44440

[17] Hotez PJ, Aksoy S, Brindley PJ, Kamhawi S. World neglected tropical diseases day. PLoS Neglected Tropical Diseases. 2020;14: 1–4. doi: 10.1371/journal.pntd.0007999 31995572 PMC6988912

[18] Stuyver LJ, Levecke B. The role of diagnostic technologies to measure progress toward WHO 2030 targets for soil-transmitted helminth control programs. PLoS Neglected Tropical Diseases. 2021;15. e0009422. doi: 10.1371/journal.pntd.0009422 34081694 PMC8174700

[19] Montresor A, Mupfasoni D, Mikhailov A, Mwinzi P, Lucianez A, Jamsheed M, et al. The global progress of soil-transmitted helminthiases control in 2020 and World Health Organization targets for 2030. PLoS Negl Trop Dis. 2020; 14: e0008505. doi: 10.1371/journal.pntd.0008505 32776942 PMC7446869

[20] WHO. Guideline: preventive chemotherapy to control soil-transmitted helminth infections in at-risk population groups. Geneva. World Health Organization; 2017. Available from: https://apps.who.int/iris/handle/10665/258983 29578660

[21] Adegnika AA, Agnandji ST, Chai SK, Ramharter M, Breitling L, Kendjo E, et al. Increased prevalence of intestinal helminth infection during pregnancy in a Sub-Saharan African community. Wien Klin Wochenschr. 2007; 119:712–716. doi: 10.1007/s00508-007-0907-z 18157604

[22] ESPEN. Equatorial Guinea. WHO. 2022. Available from: https://espen.afro.who.int/countries/equatorial-guinea

[23] Benito A, Roche J. Prevalence of intestinal parasite infections with special reference to Entamoeba histolytica on the island of Bioko (Equatorial Guinea). The American Journal of Tropical Medicine and Hygiene. 1999; 60:257–262. doi: 10.4269/ajtmh.1999.60.257 10072147

[24] WHO. APOC and Tchuem Tchuenté Louis-Albert, Wanji DS. Trazado integrado de enfermedades tropicales descuidadas en Guinea Ecuatorial: oncocercosis, filariasis linfática, loiasis, esquistosomiasis y helmintiasis Transmitida por contacto con el Suelo. Programa Africano para el Control de la Oncocercosis. 2008. Available from: https://apps.who.int/iris/handle/10665/363234

[25] Quintero Pérez W, Linares Guerra M, Téllez Almirall O, Díaz Cabrera JC, del Valle Viera M. Parasitismo intestinal en una escuela primaria de BATA, Guinea Ecuatorial. Revista de Ciencias Médicas de Pinar del Río. 2008;12:73–80.

[26] Roka M, Goñi P, Rubio E, Clavel A. Prevalence of intestinal parasites in HIV-positive patients on the island of Bioko, Equatorial Guinea: Its relation to sanitary conditions and socioeconomic factors. Science of The Total Environment. 2012; 432:404–411. doi: 10.1016/j.scitotenv.2012.06.023 22771815

[27] Roka M, Goñi P, Rubio E, Clavel A. Intestinal parasites in HIV-seropositive patients in the Continental Region of Equatorial Guinea: Its relation with socio-demographic, health and immune systems factors. Transactions of the Royal Society of Tropical Medicine and Hygiene. 2013; 107:502–510. doi: 10.1093/trstmh/trt049 23783759

[28] Ministerio de Sanidad y Bienestar Social. Plan directeur de lutte contre les maladies tropicales négligées 2018–2022. 2018.Available from: https://espen.afro.who.int/system/files/content/resources/EQUATORIAL_GUINEA_NTD_Master_Plan_2018_2022.pdf

[29] ESPEN.WHO Afro Región. ESPEN 2020 Annual Report 2021. Available from: https://espen.afro.who.int/system/files/content/resources/ESPEN%20%202020%20Annual%20Report%20En.pdf

[30] OMS. Estrategia de cooperación resumen: Guinea Ecuatorial. Situación de la Salud. Informe 2018; 2018. Avaible from: https://apps.who.int/iris/handle/10665/137167?locale-attribute=es

[31] Ministerio de Sanidad y Bienestar Social, Ministerio de Economía, Planificación e Inversiones públicas. Demographic and Health Survey (DHS) in Equatorial Guinea. 2012. Available from: https://dhsprogram.com/pubs/pdf/fr271/fr271.pdf

[32] ICH. Guideline For Good Clinical Practice.2016. Available from: https://database.ich.org/sites/default/files/E6_R2_Addendum.pdf

[33] WMA—The World Medical Association-WMA Declaration of Helsinki–Ethical Principles for Medical Research Involving Human Subjects. Available from: https://www.wma.net/policies-post/wma-declaration-of-helsinki-ethical-principles-for-medical-research-involving-human-subjects/10.1191/0969733002ne486xx 16010903

[34] Instituto Nacional de Estadísticas de Guinea Ecuatorial. Anuario Estadístico de Guinea Ecuatorial 2018. 2019. Available from: https://www.inege.gq/wp-content/uploads/2019/03/ANUARIO-ESTADISTICO-DE-GUINEA-ECUATORIAL-2018-.pdf

[35] Charan J, Biswas T. How to Calculate Sample Size for Different Study Designs in Medical Research? Indian Journal of Psychological Medicine. 2013.35:121–126. doi: 10.4103/0253-7176.116232 24049221 PMC3775042

[36] WHO. WHO Bench aids for the diagnosis of intestinal parasites, second edition. Geneve: World Health Organization; 2019. Available from: https://www.who.int/publications/i/item/9789241515344

[37] Ngwese MM, Manouana GP, Moure PAN, Ramharter M, Esen M, Adégnika AA. Diagnostic techniques of soil-transmitted helminths: Impact on control measures. Tropical Medicine and Infectious Disease. 2020;5.93. doi: 10.3390/tropicalmed5020093 32516900 PMC7344795

[38] Knopp S, Salim N, Schindler T, Voules DAK, Rothen J, Lweno O, et al. Diagnostic accuracy of Kato-Katz, FLOTAC, Baermann, and PCR methods for the detection of light-intensity hookworm and Strongyloides stercoralis infections in Tanzania. American Journal of Tropical Medicine and Hygiene. 2014;90:535–545. doi: 10.4269/ajtmh.13-0268 24445211 PMC3945701

[39] WHO. Soil-Transmitted Helminthiases: Eliminating Soil-Transmitted Helmnthiases as a Public Health Problem in Children. Progress Report 2001–2010 and strategic plan 2011–2020. World Health Organization. 2012;1–90. https://apps.who.int/iris/handle/10665/44804

[40] Tabi ESB, Eyong EM, Akum EA, Löve J, Cumber SN. Soil-transmitted Helminth infection in the Tiko Health District, Southwest Region of Cameroon: a post-intervention survey on prevalence and intensity of infection among primary school children. Pan Afr Med J. 2018. doi: 10.11604/pamj.2018.30.74.15676 30344858 PMC6191252

[41] Dejon-agobé JC, Honkpehedji YJ, Zinsou JF, Edoa JR, Adégbitè BR, Mangaboula A, et al. Epidemiology of Schistosomiasis and Soil-Transmitted Helminth Coinfections among School children Living in Lambaréné, Gabon. The American Journal of Tropical Medicine and Hygiene. 2020;103:325–333. 32431272 10.4269/ajtmh.19-0835 PMC7356410

[42] Sartorius B, Cano J, Simpson H, Tusting LS, Marczak LB, Miller-Petrie MK, et al. Prevalence and intensity of soil-transmitted helminth infections of children in sub-Saharan Africa, 2000–18: a geospatial analysis. The Lancet Global Health. 2021;9: e52–60. doi: 10.1016/S2214-109X(20)30398-3 33338459 PMC7786448

[43] World Health Organization. Ending the neglect to attain the sustainable development goals: a sustainability framework for action against neglected tropical diseases 2021–2030. Geneva: World Health Organization; 2021. Available from: https://apps.who.int/iris/handle/10665/338886

[44] Ncogo P, Herrador Z, Romay-Barja M, García-Carrasco E, Nseng G, Berzosa P, et al. Malaria prevalence in Bata district, Equatorial Guinea: a cross-sectional study. Malaria Journal. 2015; 14:456. https://malariajournal.biomedcentral.com/articles/10.1186/s12936-015-0986-7 26573911 10.1186/s12936-015-0986-7 PMC4647797

[45] Mackinnon E, Ayah R, Taylor R, Owor M, Ssempebwa J, Olago l. D, et al. 21st century research in urban WASH and health in sub-Saharan Africa: methods and outcomes in transition. International Journal of Environmental Health Research. 2019;29:457–478. doi: 10.1080/09603123.2018.1550193 30545246

[46] Kabore A, Ibikounle M, Tougoue JJ, Mupoyi S, Ndombe M, Shannon S, et al. Initiating NTD programs targeting schistosomiasis and soil-transmitted helminthiasis in two provinces of the Democratic Republic of the Congo: Establishment of baseline prevalence for Mass Drug Administration. Acta Tropica. 2017; 166:177–185. doi: 10.1016/j.actatropica.2016.11.023 27888125

[47] Bopda J, Nana-Djeunga H, Tenaguem J, Kamtchum-Tatuene J, Gounoue-Kamkumo R, Assob-Nguedia C, et al. Prevalence and intensity of human soil transmitted helminth infections in the Akonolinga health district (Centre Region, Cameroon): Are adult hosts contributing in the persistence of the transmission? Parasite Epidemiology and Control. 2016;1:199–204. doi: 10.1016/j.parepi.2016.03.001 29988185 PMC5991827

[48] Nguema RM, Mavoungou JF, Me Ngou-Milama KM, Mamfoumbi MM, Koumba AA, Lamine MS, et al. Baseline mapping of schistosomiasis and soil transmitted helminthiasis in the northern and eastern health regions of Gabon, Central Africa: Recommendations for preventive chemotherapy. Tropical Medicine and Infectious Disease. 2018; 3:119. doi: 10.3390/tropicalmed3040119 30423901 PMC6306699

[49] Zerdo Z, Bastiaens H, Anthierens S, Massebo F, Masne M, Biresaw G, et al. Prevalence, intensity and endemicity of intestinal schistosomiasis and soil-transmitted helminthiasis and its associated factors among school-aged children in Southern Ethiopia. Sci Rep. 2022; 12:4586. doi: 10.1038/s41598-022-08333-7 35302056 PMC8931111

[50] Aung E, Han KT, Gordon CA, Hlaing NN, Aye MM, Htun MW, et al. High prevalence of soil-transmitted helminth infections in Myanmar schoolchildren. Infect Dis Poverty. 2022; 11:28. doi: 10.1186/s40249-022-00952-6 35272701 PMC8908594

[51] Pullan RL, Brooker SJ. The global limits and population at risk of soil-transmitted helminth infections in 2010. Parasites and Vectors. 2012;5:1–14. 22537799 10.1186/1756-3305-5-81 PMC3419672

[52] Ndamukong Nyanga J, Kimbi H, Sumbele I, Nana Y, Bertek S, Ndamukong K, et al. A Crosssectional Study on the Influence of Altitude and Urbanisation on Co-infection of Malaria and Soiltransmitted Helminths in Fako Division, Southwest Cameroon. International Journal of TROPICAL DISEASE & Health. 2015; 8:150–164.

[53] Taylor-Robinson DC, Maayan N, Donegan S, Chaplin M, Garner P. Public health deworming programmes for soil-transmitted helminths in children living in endemic areas. Cochrane Database of Systematic Reviews. 2019. doi: 10.1002/14651858.CD000371.pub7 31508807 PMC6737502

[54] WHO. Soil-transmitted helminth infections. 2022. Available from: https://www.who.int/news-room/fact-sheets/detail/soil-transmitted-helminth-infections

[55] WHO. 2030 targets for soil-transmitted helminthiases control programmes. Geneva: World Health Organization. 2020. Available from: https://apps.who.int/iris/handle/10665/330611

[56] Manuel M, Ramanujam K, Ajjampur SSR. Molecular Tools for Diagnosis and Surveillance of Soil-Transmitted Helminths in Endemic Areas. Parasitologia. 2021; 1:105–118. 10.3390/parasitologia1030012

[57] Fenta A, Hailu T, Alemu M, Nibret E, Amor A, Munshea A. Evaluating the performance of diagnostic methods for soil transmitted helminths in the Amhara National Regional State, Northwest Ethiopia. BMC Infectious Diseases. 2020; 2:803. doi: 10.1186/s12879-020-05533-2 33121458 PMC7597015

