# Supplementary material for: Prevalence, intensity and associated risk factors of soil-transmitted helminth infections among individuals living in Bata district, Equatorial Guinea
Source: PLoS Negl Trop Dis. 2023 May 17;17(5):e0011345. doi: 10.1371/journal.pntd.0011345 (PMC10228798; doi:10.1371/journal.pntd.0011345)
Supplement: S1 Checklist — (DOC) [file pntd.0011345.s001.doc]

STROBE Statement—Checklist of items that should be included in reports of ***cross-sectional studies***

|  | Item No | Recommendation |
| --- | --- | --- |
| **Title and abstract** | 1 | (*a*) Indicate the study’s design with a commonly used term in the title or the abstract  **We indicated the study design in the abstract** |
|  |  | (*b*) Provide in the abstract an informative and balanced summary of what was done and what was found  **We described the study method and main finding in the “Methods” and ”Results” sections of the abstract** |
| Introduction | | |
| Background/rationale | 2 | Explain the scientific background and rationale for the investigation being reported  **The Scientific background and rationale for investigation are described in “Introduction” section** |
| Objectives | 3 | State specific objectives, including any prespecified hypotheses  **Study main objective is described in “Introduction section, paragraph 4”** |
| Methods | | |
| Study design | 4 | Present key elements of study design early in the paper  **Study design key elements are presented in “Materials and methods” section, already in the second sub-section** |
| Setting | 5 | Describe the setting, locations, and relevant dates, including periods of recruitment, exposure, follow-up, and data collection  **We described the setting, locations and relevant dates of the study in the “Materials and methods” section, particulary in the “study area and study population” and “study design” sub-sections** |
| Participants | 6 | (*a*) Give the eligibility criteria, and the sources and methods of selection of participants  **Eligibility criteria and methods of participant’s selection are described in in “Study design and study population” “sampling procedure” sub-sections of the “Material and methods” section.** |
| Variables | 7 | Clearly define all outcomes, exposures, predictors, potential confounders, and effect modifiers. Give diagnostic criteria, if applicable  **We defined study outcomes and exposure in “the statistical consideration” sub-section of “Materials and methods” section** |
| Data sources/ measurement | 8* | For each variable of interest, give sources of data and details of methods of assessment (measurement). Describe comparability of assessment methods if there is more than one group  **We described data sources and detailed “the statistical consideration” sub-section of “Materials and methods” section** |
| Bias | 9 | Describe any efforts to address potential sources of bias  **We developed a sampling procedure for the participants’ selection to avoid selection bias. The procedure is described in the “sampling procedure” sub-section of “Materials and methods” section.** |
| Study size | 10 | Explain how the study size was arrived at  **We explained how study size was arrived at the “sample size calculation” sub-section of the “Materials and methods” section.** |
| Quantitative variables | 11 | Explain how quantitative variables were handled in the analyses. If applicable, describe which groupings were chosen and why  **We described how quantitative variables were handled in the “Statistical consideration” sub-section of the “Materials and methods” section,** |
| Statistical methods | 12 | (*a*) Describe all statistical methods, including those used to control for confounding  **All statistical analysis methods are described in the “Statistical considerations” sub-section of the “Materials and methods” section.** |
|  |  | (*b*) Describe any methods used to examine subgroups and interactions |
|  |  | (*c*) Explain how missing data were addressed |
|  |  | (*d*) If applicable, describe analytical methods taking account of sampling strategy |
|  |  | (*e*) Describe any sensitivity analyses |
| Results | | |
| Participants | 13* | (a) Report numbers of individuals at each stage of study—eg numbers potentially eligible, examined for eligibility, confirmed eligible, included in the study, completing follow-up, and analysed  **The number of individuals at each study stage are reported in participants enrolment flow in “Results” section,(Figure .2)** |
|  |  | (b) Give reasons for non-participation at each stage  **Reason for non-participation in the study for some participants are provided in participants enrolment flow in “Results” section** |
|  |  | (c) Consider use of a flow diagram  **We used a flow diagram to describe participants flow in the study (Figure .2).** |
| Descriptive data | 14* | (a) Give characteristics of study participants (eg demographic, clinical, social) and information on exposures and potential confounders  **Study participants characteristics are described in the “study population characteristics” sub-section of the “Results” section in table 1.** |
|  |  | (b) Indicate number of participants with missing data for each variable of interest |
| Outcome data | 15* | Report numbers of outcome events or summary measures  **Summary measures are reported in the “Result” section** |
| Main results | 16 | (*a*) Give unadjusted estimates and, if applicable, confounder-adjusted estimates and their precision (eg, 95% confidence interval). Make clear which confounders were adjusted for and why they were included  **We presented unadjusted estimates and adjusted estimates and their confidence interval at the “Factor associated with STH infections” sub-section of “Results” section** |
|  |  | (*b*) Report category boundaries when continuous variables were categorized  **We categorized the age variable and we reported the category boundaries in Table 1** |
|  |  | (*c*) If relevant, consider translating estimates of relative risk into absolute risk for a meaningful time period |
| Other analyses | 17 | Report other analyses done—eg analyses of subgroups and interactions, and sensitivity analyses |
| Discussion | | |
| Key results | 18 | Summarise key results with reference to study objectives  **Key results are summarized concerning the study objective at the beginning of each paragraph of the “Discussion” section.** |
| Limitations | 19 | Discuss limitations of the study, taking into account sources of potential bias or imprecision. Discuss both direction and magnitude of any potential bias  **Study limitations are discussed in the “Discussion” section, paragraph 6** |
| Interpretation | 20 | Give a cautious overall interpretation of results considering objectives, limitations, multiplicity of analyses, results from similar studies, and other relevant evidence  **We interpreted our results based on the study objectives through the “Discussion” section. We compared our results with similar studies and we discussed the limitations of our methods.** |
| Generalizability | 21 | Discuss the generalizability (external validity) of the study results  **We discussed the generalizability of our results at the end of the “Discussion” section.** |
| Other information | | |
| Funding | 22 | Give the source of funding and the role of the funders for the present study and, if applicable, for the original study on which the present article is based  **Source of funding and roll of founders have been provided.** |

*Give information separately for exposed and unexposed groups.

**Note:** An Explanation and Elaboration article discusses each checklist item and gives methodological background and published examples of transparent reporting. The STROBE checklist is best used in conjunction with this article (freely available on the Web sites of PLoS Medicine at http://www.plosmedicine.org/, Annals of Internal Medicine at http://www.annals.org/, and Epidemiology at http://www.epidem.com/). Information on the STROBE Initiative is available at www.strobe-statement.org.
