# Supplementary material for: Prevalence, intensity and associated risk factors of soil-transmitted helminth infections among individuals living in Bata district, Equatorial Guinea
Source: PLoS Negl Trop Dis. 2023 May 17;17(5):e0011345. doi: 10.1371/journal.pntd.0011345 (PMC10228798; doi:10.1371/journal.pntd.0011345)
Supplement: S1 Table — (DOCX) [file pntd.0011345.s003.docx]

**S1 Table.** WHO classification of the intensity of *Ascaris lumbricoides*, *Trichuris trichiura*, and hookworm infections [1].

| **Infection intensity** | **Ascariasis (epg*)** | **Trichuriasis (epg)** | **Hookworm infection (epg)** |
| --- | --- | --- | --- |
| **Light** | 1 to 4 999 | 1 to 999 | 1 to 1 999 |
| **Moderate** | 5 000 to 49 999 | 1 000 to 9 999 | 2 000 to 3 999 |
| **Heavy** | ≥ 50 000 | ≥ 10 000 | ≥ 4 000 |

*egg per gram

**Reference**

1. WHO. Soil-Transmitted Helminthiases: Eliminating Soil-Transmitted Helminthiases as a Public Health Problem in Children. Progress Report 2001-2010 and strategic plan 2011-2020.World

Health Organization. 2012;1–90. https://apps.who.int/iris/handle/10665/44804
