# Supplementary material for: Prevalence, intensity and associated risk factors of soil-transmitted helminth infections among individuals living in Bata district, Equatorial Guinea
Source: PLoS Negl Trop Dis. 2023 May 17;17(5):e0011345. doi: 10.1371/journal.pntd.0011345 (PMC10228798; doi:10.1371/journal.pntd.0011345)
Supplement: S2 Table — Table A. Assessment of the multi-collinearity using the Variance Inflation Factor (VIF) in the initial final model of the multivariable analysis Table B. Assessment of the multi-collinearity using the Variance Inflation Factor (VIF) in the revised final model of the multivariate analysis. (DOCX) [file pntd.0011345.s004.docx]

**Table A in S2 Table**. Assessment of the multi-collinearity using the Variance Inflation Factor (VIF) in the initial final model of the multivariable analysis

| Variable | VIF | 95%CI(VIF) | Increased SE | Tolerance | 95%CI (Tolerance) |
| --- | --- | --- | --- | --- | --- |
| Age group | 5.88 | 4.89 – 7.13 | 2.43 | 0.17 | 0.14 – 0.20 |
| Gender | 1.09 | 1.03 – 1.35 | 1.05 | 0.91 | 0.74 – 0.98 |
| Locality | 1.88 | 1.63 – 2.21 | 1.37 | 0.53 | 0.45 – 0.61 |
| Education | 3.99 | 3.35 – 4.81 | 2.00 | 0.25 | 0.21 – 0.30 |
| Occupation | 9.12 | 7.52 – 11.12 | 3.02 | 0.11 | 0.09 – 0.13 |
| Type of toilet | 2.14 | 1.84 – 2.53 | 1.46 | 0.47 | 0.39 – 0.54 |
| Type of toilet floor | 1.58 | 1.39 – 1.85 | 1.26 | 0.63 | 0.54 – 0.72 |

**Table B in S2 Table.** Assessment of the multi-collinearity using the Variance Inflation Factor (VIF) in the revised final model of the multivariate analysis

| Variable | VIF | 95%CI(VIF) | Increased SE | Tolerance | 95%CI (Tolerance) |
| --- | --- | --- | --- | --- | --- |
| Age group | 2.21 | 1.90 – 2.62 | 1.49 | 0.45 | 0.38 – 0.53 |
| Gender | 1.07 | 1.01 – 1.38 | 1.04 | 0.93 | 0.73 – 0.99 |
| Locality | 1.70 | 1.49 – 2.00 | 1.30 | 0.59 | 0.50 – 0.67 |
| Education | 2.08 | 1.80 – 2.47 | 1.44 | 0.48 | 0.41 – 0.56 |
| Type of toilet | 2.02 | 1.74 – 2.39 | 1.42 | 0.50 | 0.42 – 0.57 |
| Type of toilet floor | 1.55 | 1.37 – 1.82 | 1.24 | 0.65 | 0.55 – 0.73 |
