# Supplementary material for: Prevalence, intensity and associated risk factors of soil-transmitted helminth infections among individuals living in Bata district, Equatorial Guinea
Source: PLoS Negl Trop Dis. 2023 May 17;17(5):e0011345. doi: 10.1371/journal.pntd.0011345 (PMC10228798; doi:10.1371/journal.pntd.0011345)
Supplement: S3 Table — (DOCX) [file pntd.0011345.s005.docx]

**S3 Table.** Distribution per education level, occupation, and household characteristics of soil-transmitted helminths (STH) infection among the study population

|  | | N | Any STH infection | | |  | *Ascaris lumbricoides* | | | |  | | *Trichuris trichiura* | | |  | Hookworm | | |
| --- | --- | --- | --- | --- | --- | --- | --- | --- | --- | --- | --- | --- | --- | --- | --- | --- | --- | --- | --- |
|  |  |  | **n** | **%** | **95%CI (%)** |  | **n** | **%** | **95%CI (%)** |  | | **n** | | **%** | **95%CI (%)** |  | **n** | **%** | **95%CI (%)** |
| Education | |  |  |  |  |  |  |  |  |  | |  | |  |  |  |  |  |  |
|  | No education | 91 | 47 | 51.6 | 41.0 – 62.2 |  | 36 | 39.6 | 29.6 – 50.4 |  | | 23 | | 25.3 | 17.0 – 35.7 |  | 1 | 1.1 | 0.1 – 6.8 |
|  | Primary | 188 | 126 | 67.0 | 59.7 – 73.6 |  | 87 | 46.3 | 39.0 – 53.7 |  | | 94 | | 50.0 | 42.9 – 57.1 |  | 9 | 4.8 | 2.4 – 9.2 |
|  | Secondary/University | 60 | 31 | 51.7 | 38.5 – 64.6 |  | 22 | 36.7 | 24.9 – 50.2 |  | | 20 | | 33.3 | 22.0 – 46.8 |  | 3 | 5.0 | 1.3 – 14.8 |
| Daily occupation | |  |  |  |  |  |  |  |  |  | |  | |  |  |  |  |  |  |
|  | Student | 168 | 116 | 69.0 | 61.4 – 75.8 |  | 82 | 48.8 | 41.1 – 56.6 |  | | 88 | | 52.4 | 44.6 – 60.1 |  | 7 | 4.2 | 1.8 – 8.7 |
|  | Farmer/fisher | 65 | 36 | 55.4 | 42.6 – 67.5 |  | 26 | 40.0 | 28.3 – 52.9 |  | | 19 | | 29.2 | 18.9 – 42.0 |  | 5 | 7.7 | 2.9 – 17.8 |
|  | Unemployed | 62 | 31 | 50.0 | 37.0 – 63.0 |  | 22 | 35.5 | 24.0 – 48.7 |  | | 21 | | 33.9 | 22.6 – 46.8 |  | 0 | 0.0 | 0.0 – 7.3 |
|  | Others | 45 | 21 | 46.7 | 31.7 – 62.1 |  | 15 | 33.3 | 20.0 – 48.9 |  | | 9 | | 20.0 | 10.1 – 34.6 |  | 1 | 2.2 | 0.0 – 11.8 |
| House type | |  |  |  |  |  |  |  |  |  | |  | |  |  |  |  |  |  |
|  | Cement | 128 | 76 | 59.4 | 50.3 – 67.9 |  | 52 | 40.6 | 32.1 – 49.7 |  | | 52 | | 40.6 | 32.1 – 49.7 |  | 4 | 3.1 | 1.0 – 8.3 |
|  | Wood | 184 | 108 | 58.7 | 51.2 – 65.8 |  | 80 | 43.5 | 36.3 – 51.0 |  | | 70 | | 38.0 | 31.1 – 45.5 |  | 8 | 4.3 | 2.0 – 8.7 |
|  | Mixed | 28 | 20 | 71.4 | 51.1 – 86.0 |  | 13 | 46.4 | 28.0 – 65.8 |  | | 15 | | 53.6 | 34.2 – 72.0 |  | 1 | 3.6 | 0.2 – 20.2 |
| House floor type | |  |  |  |  |  |  |  |  |  | |  | |  |  |  |  |  |  |
|  | Cemented | 290 | 173 | 59.7 | 53.7 – 65.3 |  | 123 | 42.4 | 36.7 – 48.3 |  | | 116 | | 40.0 | 34. 4 – 45.9 |  | 11 | 3.8 | 2.0 – 6.9 |
|  | Non cemented | 50 | 31 | 62.0 | 47.2 – 75.0 |  | 22 | 44.0 | 30.3 – 58.7 |  | | 21 | | 42.0 | 28. 5 – 56.7 |  | 2 | 4.0 | 0.7 – 14.9 |
| Availability of toilets | |  |  |  |  |  |  |  |  |  | |  | |  |  |  |  |  |  |
|  | Available | 325 | 193 | 59.4 | 53.8 – 64.7 |  | 135 | 41.5 | 36.2 – 47.1 |  | | 132 | | 40.6 | 35.3 – 46.2 |  | 13 | 4.0 | 2.2 – 6.9 |
|  | Not available | 15 | 11 | 73.3 | 44.8 – 91.1 |  | 10 | 66.7 | 38.7 – 87.0 |  | | 5 | | 33.3 | 13.0 – 61.3 |  | 0 | 0.0 | 0.0 – 25.3 |
| Type of toilet | |  |  |  |  |  |  |  |  |  | |  | |  |  |  |  |  |  |
|  | Private /modern | 156 | 81 | 51.9 | 43.8 – 59.9 |  | 55 | 35.3 | 27.9 – 43.4 |  | | 53 | | 34.0 | 26.7 – 42.0 |  | 5 | 3.2 | 1.2 – 7.7 |
|  | Communal latrine | 64 | 39 | 60.9 | 47.9 – 72.6 |  | 27 | 42.2 | 30.2 – 55.2 |  | | 29 | | 45.3 | 33.0 – 58.2 |  | 3 | 4.7 | 1.2 – 14.0 |
|  | Traditional latrine/Hole | 105 | 73 | 69.5 | 59.7 – 77.9 |  | 53 | 50.5 | 40.6 – 60.3 |  | | 50 | | 47.6 | 37.9 – 57.5 |  | 5 | 4.8 | 1.8 – 11.3 |
| Type of toilet floor | |  |  |  |  |  |  |  |  |  | |  | |  |  |  |  |  |  |
|  | Cemented | 268 | 154 | 57.5 | 51.3 – 63.4 |  | 109 | 40.7 | 34.8 – 46.8 |  | | 104 | | 38.8 | 33.0 – 44.9 |  | 9 | 3.4 | 1.6 – 6.5 |
|  | Non-cemented | 57 | 39 | 68.4 | 54.6 – 79.7 |  | 26 | 45.6 | 32.6 – 59.2 |  | | 28 | | 49.1 | 35.8 – 62.6 |  | 4 | 7.0 | 2.3 – 17.8 |
| Water source | |  |  |  |  |  |  |  |  |  | |  | |  |  |  |  |  |  |
|  | Tap | 189 | 112 | 59.3 | 51.9 – 66.3 |  | 76 | 40.2 | 33.2 – 47.6 |  | | 82 | | 43.4 | 36.3 – 50.8 |  | 8 | 4.2 | 2.0 – 8.5 |
|  | Well | 89 | 54 | 60.7 | 49.7 – 70.7 |  | 42 | 47.2 | 36.6 – 58.0 |  | | 33 | | 37.1 | 27.3 – 48.0 |  | 5 | 5.6 | 2.1 – 13.2 |
|  | River | 62 | 38 | 61.3 | 48.0 – 73.1 |  | 27 | 43.5 | 31.2 – 56.7 |  | | 22 | | 35.5 | 24.0 – 48.7 |  | 0 | 0.0 | 0.0 – 7.3 |
| Sewage system | |  |  |  |  |  |  |  |  |  | |  | |  |  |  |  |  |  |
|  | Available | 14 | 9 | 64.3 | 35.6 – 86.0 |  | 6 | 42.9 | 18.8 – 70.4 |  | | 3 | | 21.4 | 5.7 – 51.2 |  | 0 | 0.0 | 0.0 – 26.8 |
|  | Not Available | 326 | 195 | 59.8 | 54.3 – 65.1 |  | 139 | 42.6 | 37.2 – 48.2 |  | | 134 | | 41.1 | 35.7 – 46.7 |  | 13 | 4.0 | 2.2 – 6.9 |
